# Isoflavones inhibit intestinal epithelial cell proliferation and induce apoptosis in vitro

**DOI:** 10.1038/sj.bjc.6690559

**Published:** 1999-07

**Authors:** C Booth, D F Hargreaves, J A Hadfield, A T McGown, C S Potten

**Affiliations:** Epithelial Biology Group, CRC Section of Cell and Tumour Biology, CRC Section of Drug Discovery and Imaging, Paterson Institute for Cancer Research, Christie Hospital NHS Trust, Wilmslow Road, Manchester M20 4BX, UK

**Keywords:** genistein, isoflavones, epithelial cell, intestine, apoptosis

## Abstract

There have been many reports that high soya-based diets reduce the risk of certain types of cancer. This effect may be due to the presence of high levels of isoflavones derived from the soya bean, particularly genistein which has been shown to be a protein tyrosine kinase (PTK) inhibitor and have both oestrogenic and anti-oestrogenic properties. We have examined the effect of genistein and a number of novel synthetic analogues on both normal (IEC6, IEC18) and transformed (SW620, HT29) intestinal epithelial cell lines. Responses were compared to those elicited by oestradiol, the anti-oestrogen tamoxifen, and the tyrosine kinase inhibitor tyrphostin. Genistein and tamoxifen were potent inhibitors of cell proliferation. Of seven novel isoflavones tested, none were more potent inhibitors than genistein, and all displayed similar relative activities across the different cell lines. In addition to inhibiting cell proliferation, cell death via apoptosis was observed when the cells were exposed to the isoflavones and all but one exhibited PTK inhibitory activity. These data suggest that by reducing proliferation and inducing apoptosis, possibly due in part to PTK inhibition, isoflavones may have a role in protecting normal intestinal epithelium from tumour development (reducing the risk) and may reduce colonic tumour growth. © 1999 Cancer Research Campaign


*British Journal of Cancer* (1999) **80**(10), 1550–1557
© 1999 Cancer Research Campaign
Article no. bjoc.1999.0559

### Isoflavones inhibit intestinal epithelial cell proliferation and induce apoptosis in vitro

**C Booth[1], DF Hargreaves[1], JA Hadfield[2], AT McGown[2] and CS Potten[1]**

[1]Epithelial Biology Group, CRC Section of Cell and Tumour Biology, [2]CRC Section of Drug Discovery and Imaging, Paterson Institute for Cancer Research, Christie Hospital NHS Trust, Wilmslow Road, Manchester M20 4BX, UK

**Summary** There have been many reports that high soya-based diets reduce the risk of certain types of cancer. This effect may be due to the presence of high levels of isoflavones derived from the soya bean, particularly genistein which has been shown to be a protein tyrosine kinase (PTK) inhibitor and have both oestrogenic and anti-oestrogenic properties. We have examined the effect of genistein and a number of novel synthetic analogues on both normal (IEC6, IEC18) and transformed (SW620, HT29) intestinal epithelial cell lines. Responses were compared to those elicited by oestradiol, the anti-oestrogen tamoxifen, and the tyrosine kinase inhibitor tyrphostin. Genistein and tamoxifen were potent inhibitors of cell proliferation. Of seven novel isoflavones tested, none were more potent inhibitors than genistein, and all displayed similar relative activities across the different cell lines. In addition to inhibiting cell proliferation, cell death via apoptosis was observed when the cells were exposed to the isoflavones and all but one exhibited PTK inhibitory activity. These data suggest that by reducing proliferation and inducing apoptosis, possibly due in part to PTK inhibition, isoflavones may have a role in protecting normal intestinal epithelium from tumour development (reducing the risk) and may reduce colonic tumour growth.

**Keywords:** genistein; isoflavones; epithelial cell; intestine; apoptosis

The incidence of intestinal cancer is high in Western populations but is relatively uncommon in Asia and Africa (Boyle et al, 1985). It is thought that this variation in incidence is strongly influenced by diet (Armstrong and Doll, 1975; Adlercreutz, 1990) and it is estimated that about 32% of deaths by all cancers, and 70% of deaths from colorectal cancer, could be prevented by a change in diet (Willet, 1995). There is also evidence to suggest that a high intake of soya-based foods (as in populations of Southeast Asia) could contribute to a reduced incidence of mammary (Lee et al, 1995) and prostate (Severson et al 1989) cancer.

Soya beans are an abundant source of the isoflavones genistein, daidzein and glyceitin (Wang and Murphy, 1994). These diphenol molecules are structurally similar to the steroidal oestrogens and thus, have been termed phyto-oestrogens. Due to this similarity, attention has focused on the role of phyto-oestrogens in preventing both hormonally mediated (particularly breast) and hormonally independent cancers. Soya metabolites may reduce the risk of colon cancers (Messina et al, 1994). Genistein in particular possesses several anti-tumorigenic activities in vitro, including inhibition of angiogenesis (Fotsis et al, 1993), topoisomerase (Okura et al, 1988) and tyrosine kinase (Ogawara et al, 1989) activity, in addition to having antioxidant properties (Wei et al, 1993). Experiments using colon cancer cell lines have shown that genistein, and the related isoflavone biochanin A, can inhibit cell proliferation and induce apoptosis (Yanagihara et al, 1993; Kuo, 1996), again implying protective effects.

No data are available to demonstrate the effects of isoflavones on normal intestinal cell lines. In this study we aimed to investigate the proliferative and apoptotic effects of genistein in vitro on normal intestinal epithelial cells and compare these to the effects on malignant cell lines. We also aimed to examine the effects of some novel isoflavones, which have been synthesized primarily as specific tyrosine kinase inhibitors, upon the same cell lines. The effects of these isoflavones are compared with the effects of oestradiol, the anti-oestrogen tamoxifen, and the specific tyrosine kinase inhibitor, tyrphostin.

*Received 23 July 1998*
*Revised 5 February 1999*
*Accepted 11 February 1999*

*Correspondence to:* C Booth

### MATERIALS AND METHODS

### Cell culture methods

IEC18 and IEC6 cells, related clones isolated from normal neonatal rat small intestine, were originally obtained from A Quaroni (Quaroni and May, 1980). In all assays these lines were used below passage 20. SW620 and HT29 human colon adenocarcinoma lines were obtained from the English Tissue Culture Collection (ETCC, Porton Down, Wilts, UK). All cells were maintained and passaged in Dulbecco's modified Eagles medium (DMEM) (GibcoBRL, Paisley, UK) containing 5% fetal calf serum (FCS) (Sigma, Poole, UK), 0.25 U $ml^{-1}$ insulin (Hypurin, Fisons, Loughborough, UK), 100 U $ml^{-1}$ penicillin and 30 $\mu g\ ml^{-1}$ streptomycin (all Sigma, Poole, UK). Charcoal-stripped FCS, phenol red free DMEM, genistein, biochanin A, oestradiol, tamoxifen and tyrphostin 25 were all purchased from Sigma. Novel isoflavones were synthesized and purified within the Paterson Institute (see below).

### Isoflavone synthesis

Synthetic intermediates were purchased from Aldrich, Kodak or Lancaster Synthesis. NMR spectra were determined on either a Hitachi Perkin-Elmer R-600 (at 60 MHz) or a Bruker AC300 (at 300 MHz) NMR spectrometer in $CDCl_3$ (unless otherwise stated) and are expressed in δ values relative to tetramethylsilane. Infrared spectra were recorded on a Mattson 1000 FTIR spectrometer and are expressed in $cm^{-1}$. Melting points are uncorrected. Microanalyses were carried out by the Micro-analytical laboratory, Department of Chemistry, University of Manchester, UK. Electron impact mass spectra were determined using a VG Trio 2 mass spectrometer at an ionization energy of 70 eV.

### 5,7-Dihydroxy-3-(2-methoxyphenyl)-4H-1-benzopyran-4-one (*compound 1*)

To a solution of 2-methoxybenzyl-2,4,6-trihydroxyphenyl ketone (1.5 g, 5.47 mmol) in dimethyl formamide (15 ml) under argon was added $BF_3$ etherate (2.7 ml, 3.12 g, 21.9 mmol) followed by methanesulphonyl chloride (1.26 ml, 1.86 g, 16.3 mmol). The mixture was heated on a steam bath in an open flask for 90 min, cooled, poured into water (60 ml) and extracted with dichloromethane (3 × 20 ml). The organic extracts were washed with water (2 × 20 ml), brine (20 ml) and dried ($MgSO_4$). After evaporation of the solvent the resultant yellow gum solidified on trituration with ether and dichloromethane. Crystallization from aqueous ethanol gave a white powder (1.23 g, 79%) mp 192–193°C (lit mp 195–197°C (16). IR (KBr): 3309 (OH); 1650 (C=O); 1621 (C=C). NMR: 3.70 (3 H, s, OMe); 6.22, 6.40 (2 H, 2 d, J = 2 Hz, 6-H, 8-H); 6.90–7.52 (4 H, m, ArH); 7.89 (1 H, s, exchanges with $D_2O$, 5-OH); 8.20 (1 H, s, C=C-H); 9.91(1 H, bs, exchanges with $D_2O$, 7-OH). $M^+$, 284 (100%); 253(M – OMe, 91%).

### 5,7-Dihydroxy-2-ethoxycarbonyl-3-(2-methoxyphenyl)-4H-1-benzopyran-4-one (*compound 2*)

To a stirred solution of 2-methoxybenzyl-2,4,6-trihydroxyphenyl ketone (1 g, 3.65 mmol) in pyridine (6.8 ml) at 0°C was added ethyl oxalyl chloride (1.63 ml, 1.99 g, 14.6 mmol). After 24 h the reaction mixture was poured into water (50 ml) and extracted with chloroform (3 × 15 ml). The combined organic extracts were washed with 2 M hychochloric acid (HCl) (3 × 10 ml), dried ($MgSO_4$) and the solvent evaporated to give the title ester (**2**) as a yellow solid (1.04 g, 80%) from aqueous methanol mp 151–2°C (lit mp 154–6°C (16). $M^+$, 356 (69%); 325 (M – OMe, 81); 283 (M – $EtCO_2$, 100).

### 3-Methoxybenzyl-2,4,6-trihydroxyphenyl ketone (*compound 3*)

A stream of dry HCl gas was passed through a solution of phloroglucinol (10 g, 79.3 mmol), 3-methoxyphenylacetonitrile (10 g, 68 mmol) and zinc chloride (4 g) in diethyl ether at 0°C for 4 h. After 2 days at 0°C, 200 ml ether was added to precipitate a yellow–orange oil. The ether was decanted and the residual oil heated under reflux in 1% aqueous sulphuric acid (500 ml) for 1 h. On cooling the ketone precipitated as colourless crystals (13.98 g, 75%), mp 165–166°C (from aqueous methanol). (Found: C, 65.9; H, 5.1 $C_{15}H_{14}O_5$ requires C, 65.7; H, 5.1%). IR (KBr): 3272 (OH); 1629 (C = O). NMR: 3.74 (3 H, s, OMe); 4.32 (2 H, s, $ArCH_2$); 5.82 (2 H, s, 3-H, 5-H); 6.82 (3 H, bs, 2-H, 3-H, 4-H); 7.16 (1 H, d, J = 7 Hz, 5-H); 10.40, 12.20 (3 H, 2bs, exchange with $D_2O$, 3 × OH).

### 5,7-Dihydroxy-3-(3-methoxyphenyl)-4H-1-benzopyran-4-one (*compound 4*)

From ketone (**3**) (1 g, 3.65 mmol) by the method described above for isoflavone (**1**) was obtained the title compound (**4**) as white crystals (0.86 g, 83%) mp 190–2°C (from aqueous ethanol). (Found: C, 67.65; H, 4.2. $C_{16}H_{12}O_5$ requires C, 67.6; H, 4.3%). IR (KBr): 3199 (OH); 1653 (C=O). NMR: 3.74 (3 H, s, OMe); 6.20, 6.32 (2 H, 2 d, J = 2 Hz, 6-H, 8-H); 6.88–7.50 (4 H, m, 2,4,5, 6-Hs); 7.83 (1 H, s, exchanges with $D_2O$, 5-OH); 8.37 (1 H, s, 2-H); 9.80–9.95 (1 H, bs, exchanges with $D_2O$, 7-OH). $M^+$, 284 (100%).

### 5,7-Dihydroxy-3-(3-hydroxyphenyl)-4H-1-benzopyran-4-one (*compound 5*)

A solution of methyl ether (**4**) (200 mg, 0.7 mmol) in hydrobromic acid (2.3 ml, d = 1.46) and acetic acid (2.3 ml) was heated at 140–150°C for 3 h. After cooling, water (5 ml) was added and the product was filtered off as a fawn solid (180 mg, 95%) mp 242°C (lit mp 203–205°C, (17). (Found: C, 63.0; H, 4.2. $C_{15}H_{10}O_5$. $H_2O$ requires C, 62.5; H, 4.2%). NMR (acetone-$d_6$): 6.40 (1 H, d, J = 2 Hz, 6-H); 6.53 (1 H, d, J = 2 Hz, 8-H); 6.97 (1 H, dd, J = 8 Hz, J = 2 Hz, 4-H); 7.14 (1 H, bd, J = 8 Hz, 6-H); 7.23 (1 H, dd, J = 3 Hz, J = 2 Hz, 2-H); 7.36 (1 H, t, J = 8 Hz, 5-H); 8.30 (1 H, s, 2-H), 9.70–10.00 (2 H, bs, 3-OH, 7-OH); 13.07 (1 H, s, 5-OH). $M^+$, 270 (100%).

### 3,4,5-Trimethoxybenzyl-2,4,6-trihydroxyphenyl ketone (*compound 6*)

By the method described for ketone (**3**) from 3,4,5-trimethoxyphenylacetonitrile (5 g, 23.2 mmol), phloroglucinol (5 g, 39.7 mmol) and zinc chloride (4 g) in ether (75 ml) the title ketone (**6**) was obtained as colourless crystals (5.05 g, 63%) mp 197–198°C (from aqueous methanol). (Found: C, 61.2; H, 5.6. $C_{17}H_{18}O_7$ requires C, 61.1; H, 5.4%). IR (KBr): 3411, 3280 (OH); 1640 (C=O). NMR (DMSO-$d_6$): 3.30 (1 H, bs, exchanges with $D_2O$, OH); 3.75, 3.83 (9 H, 2 s, 3 × OMe); 4.28 (2 H, s, $CH_2$); 5.84 (2 H, s, 3,5-Hs); 6.54 (2 H, s, 2,6-Hs); 8.60 (2 H, bs, exchanges with $D_2O$, 2 × OH). $M^+$, 334 (33%); 153 (M – 3,4,5-triMeOphenyl$CH_2$, 100%).

### 5,7-Dihydroxy-3-(3,4,5-trimethoxyphenyl)-4H-1-benzopyran-4-one (*compound 7*)

By the method described for isoflavone (**1**) the title heterocycle (**7**) was prepared from ketone (**6**) (0.6 g, 1.8 mmol), boron trifluoride etherate (0.9 ml) and methanesulphonyl chloride (0.42 ml) as a white crystalline solid from aqueous ethanol (0.83 g, 81%) mp 224–226°C (lit mp 228°C. (Found: C, 62.2; H, 4.6. Calculated for $C_{18}H_{16}O_7$ C, 62.3; H, 4.7%). IR (KBr): 3351 (OH); 1670 (C=O). NMR: 3.68 (3 H, s, OMe); 3.78 (6 H, s, 2 × OMe); 6.20, 6.39 (2 H, 2 d, J = 2 Hz, 6, 8-Hs); 6.86 (2 H, s, 2, 6-Hs); 7.83 (1 H, s, exchanges with $D_2O$, 5-OH); 8.41 (1 H, s, 2-H); 9.80–9.95 (1 H, bs, exchanges with $D_2O$, 7-OH). $M^+$, 344 (100%); 329 (M-$CH_3$, 32).

© 1999 Cancer Research Campaign

### 5-Hydroxy-7-methoxy-3-(3-methoxyphenyl)-4H-1-benzopyran-4-one (*compound 8*)

A mixture of isoflavone **5** (200 mg, 0.7 mmol), dimethyl sulphate (1.41 mmol) and sodium hydroxide (0.062 g) in water (2 ml) was heated at 70–80°C for 1 h. The solvent was decanted and remaining solid was dried and crystallized from ethanol as white crystals (150 mg, 71%) mp 99–100°C. (Found: C, 68.4; H, 4.5. $C_{17}H_{14}O_5$ requires C, 68.5; H, 4.7%). $M^+$, 298 (100%).

### 5,7-Dihydroxy-2-ethoxycarbonyl-3-(3,4,5,-methoxyphenyl)-4H-1-benzopyran-4-one (*compound 9*)

To a stirred solution of ketone **6** (0.9 g, 2.7 mmol) in pyridine (5 ml) at 0°C was added ethyl oxalyl chloride (1.2 ml, 10.7 mmol). After stirring overnight the mixture was poured into cold water (20 ml) and extracted with chloroform (4 × 5 ml), water (5 ml) and dried over $MgSO_4$. Evaporation afforded a froth which, after trituration with ether and crystallization from aqueous ethanol, yielded the title ketone (**9**) (0.99 g, 85%) mp 199–200°C. (Found: C, 60.5; H, 4.8. $C_{21}H_{20}O_9$ requires C, 60.6; H, 4.8%). IR (KBr): 3288 (OH); 1725 (C=O, ester); 1660 (C=O, ketone). NMR: 1.01 (3 h, t, J= 7 Hz, $\underline{CH_3}CH_2$); 2.75, 2.79 (9 H, 2 s, 3 × OMe) 4.18 (2 H, q, J=7 Hz $CH_3\underline{CH_2}$); 6.32, 6.45 (2 H, 2 d, J 2 Hz, ArHs *ortho* to OHs); 6.62 (2 H, s, ArHs *ortho* to OMe). $M^+$, 416 (100%); 401 (M-OMe, 25%).

### Standard assay procedure

For both growth stimulation and inhibition assays $2 \times 10^4$ IEC6 (or IEC18) cells per well were plated out in 24-well culture plates (Costar) containing 0.2% (stimulatory assay) or 2% FCS (inhibitory assay), in 1 ml of the above media. Two per cent FCS will maintain significant cell growth, whereas 0.2% permits cell survival with minimal proliferation. Adenocarcinoma cell lines were all plated out in 2% FCS to permit optimal initial attachment.

The assay was conducted 24 h after plating ($t$ = 0) when all the cells were attached to the plates. Cells were then change into the appropriate medium with or without the freshly dissolved test substance (isoflavone). Plates were fed by addition of 0.5 ml of the appropriate media on day 3, and harvested as described below.

For assays in non-oestrogenic conditions, cells grown in standard 5% FCS DMEM were trypsinized, stopped in media containing normal serum and then washed in phenol red free media (PRFM). Cells were then plated out as above in PRFM containing either 2 or 0.2% charcoal-stripped FCS.

Each isoflavone was dissolved in dimethylsulphoxide (DMSO) to 50 mg $ml^{-1}$ and then diluted to the highest concentration (50 μg $ml^{-1}$) in media. This was sterile filtered through a 0.2 μm membrane and diluted as necessary. Slow dilution with constant vortexing maintained the isoflavones in solution. Freshly dissolved isoflavones were used for each feed because activity was found to be reduced on storage (data not shown).

Growth curves were obtained by fixing the plates at the relevant time points with 70% ethanol and after drying, staining with 0.1% crystal violet for 5 min. After rinsing with water and air-drying, the dye was extracted from the cells using 2% sodium deoxycholate and the staining intensity measured by absorption at 595 nm. Using this technique we have previously shown that absorption is an accurate index of cell number between $10^4$ and $2.5 \times 10^5$ cells per well. Outside this range responses are detectable but not linear (Potten et al, 1993).

### Tyrosine kinase assay

Stock solutions (200 ml) of the mouse leukaemic cell line P388 were prepared. Cells were collected during exponential growth ($5 \times 10^5$ cells $ml^{-1}$), washed once in cold phosphate-buffered saline (PBS) and then vortexed in cold lysis buffer (10 mM HEPES, pH 7.6, 10 μM sodium chloride (NaCl), 2 μM EDTA, 1 μM phenylmethyl sulphonyl fluoride (PMSF), 10 μg $ml^{-1}$ aprotenin and 10 μg $ml^{-1}$ leupeptin). All subsequent steps were carried out at 4°C. The lysates were homogenized in a Dounce homogenizer and nuclei and cell debris were removed by centrifugation (10 min at 1000 ***g***). Membrane extracts were then pelleted using a Beckman (USA) L8-M ultracentrifuge (30 min at $1 \times 10^5$ ***g***) and then were resuspended in HNG buffer (50 mM HEPES, pH 7.6, 125 mM NaCl and 10% (v/v) glycerol). Protein concentration was determined using Biorad protein assay with a bovine serum/albumin (BSA) standard curve. Solutions were divided into 35 μl aliquots and immediately frozen at –80°C.

Assay of PTK inhibition was based on measurement of the phosphorylation of an exogenous synthetic peptide, RR-SRC (Casnellie et al, 1982). This peptide was based on the autophosphorylation site of $pp60^{v\text{-}src}$, with two additional arginine residues at one end in order to facilitate attachment to phosphocellulose paper. Control assays with the RR-SRC peptide were used to measure non-specific phosphorylation.

Each drug was initially screened at high concentration (5 mM). If inhibition was observed then a range of concentrations were used to define an $IC_{50}$ (the drug concentration necessary to achieve 50% inhibition of phosphorylation). All drugs were dissolved in DMSO and all experiments were performed in duplicate. Each assay consisted of 10 μl standard substrate solution (1 mM RR-SRC peptide substrate, 60 mM HEPES, 20 mM magnesium chloride ($MgCl_2$), 40 μM EDTA, 200 μM dithiothreitol (DTT), 50 μg $ml^{-1}$ BSA, 0.3% (v/v) Nonidet P-40, 140 μM sodium orthovanadate and 120 μM ATP) or control solution (as substrate solution but without RR-SRC peptide), 0.5 μl [$\gamma$-$^{32}$P]-ATP, 0.5 μl PBS, 7 μl membrane solution (as prepared above, diluted to optimum concentration with PBS) and 2 μl DMSO (with or without inhibitor). Assay tubes were incubated at 30°C for 30 min, after which the reaction was terminated by addition of 20 μl ice-cold 10% TCA. After 10 min on ice membranes were precipitated by microcentrifugation, and 20 μl of supernatant per assay was spotted on individual phosphocellular discs, washed twice in 1% acetic acid and twice in water to remove non-incorporated [$\gamma$-$^{32}$P], before liquid scintillation counting. Three further vials, each containing 0.09 μl [$\gamma$-$^{32}$P]-ATP (2 μl of substrate solution mixed with [$\gamma$-$^{32}$P]-ATP and PBS) in 5 ml scintillation fluid were measured in order to calibrate the radioactivity of the [$\gamma$-$^{32}$P] for that experiment.

### Apoptosis

Apoptotic cells were identified by staining with Hoescht 33258. Briefly, 25 μl of trypsinized cells in serum-free media ($10^6$ $ml^{-1}$) were allowed to attach to each well of a poly-L-lysine coated multi-well glass slide (contained in a Petri dish) for 15 min. Fifteen millilitres of 2% FCS containing DMEM was then added to the Petri dish, thereby covering the slide. After 24 h the medium was replaced with fresh medium containing the isoflavone to be assayed. After 3 days, slides were fixed for 10 min in 4% formal saline, washed in PBS, permeabilized for 5 min in 1% Nonidet-P40 in PBS, and stained for 20 min in 10 μg $ml^{-1}$ Hoescht 33258.

 *© 1999 Cancer Research Campaign*

### RESULTS

### Effect of genistein on normal intestinal epithelial cells

Figure 1 shows that concentrations above 1 µg ml$^{-1}$ genistein dramatically inhibited cell proliferation in 2% FCS in the untransformed rat small intestinal cell line, IEC18. The same result was true for the related IEC6 cell line (data not shown). However, it can also be seen from Figure 1 that lower doses of genistein (< 1 µg ml$^{-1}$), appeared to drive a slight stimulation in cell growth (more pronounced when control growth was minimal, i.e. 0.2% FCS or growth in oestrogen-free conditions (data not shown). At high genistein concentrations (25 µg ml$^{-1}$) inhibition of cell growth was accompanied by cell death via apoptosis (Figure 2 A–C).

### Genistein and tamoxifen are more potent inhibitors of intestinal cell proliferation than their natural relatives

Biochanin A also inhibited IEC18 cell growth, although this isoflavone was less potent in its inhibitory activity than genistein (Figure 3). Oestradiol and tyrphostin possessed similar activity to biochanin A, whereas the anti-oestrogen tamoxifen was an equally, if not more potent, inhibitor than genistein. Stimulatory effects could not be induced with any of these novel isoflavones in this dose range (0.1–25 µg ml$^{-1}$). Experiments using much lower concentrations of estradiol, indicated a slight growth stimulation at $10^{-9}$ (0.27 ng ml$^{-1}$) to $10^{-11}$ M (data not shown).

This activity profile was similar in the SW620 tumour cell line (Figure 4A), although genistein did not show any stimulatory effect at low concentrations. The induction of apoptosis in HT29 cells by genistein is illustrated in Figure 2 D,E. Interestingly, in the HT29s, tamoxifen exhibited a much more marked inhibitory effect (Figure 4B) whereas oestradiol was only a weak growth inhibitor.

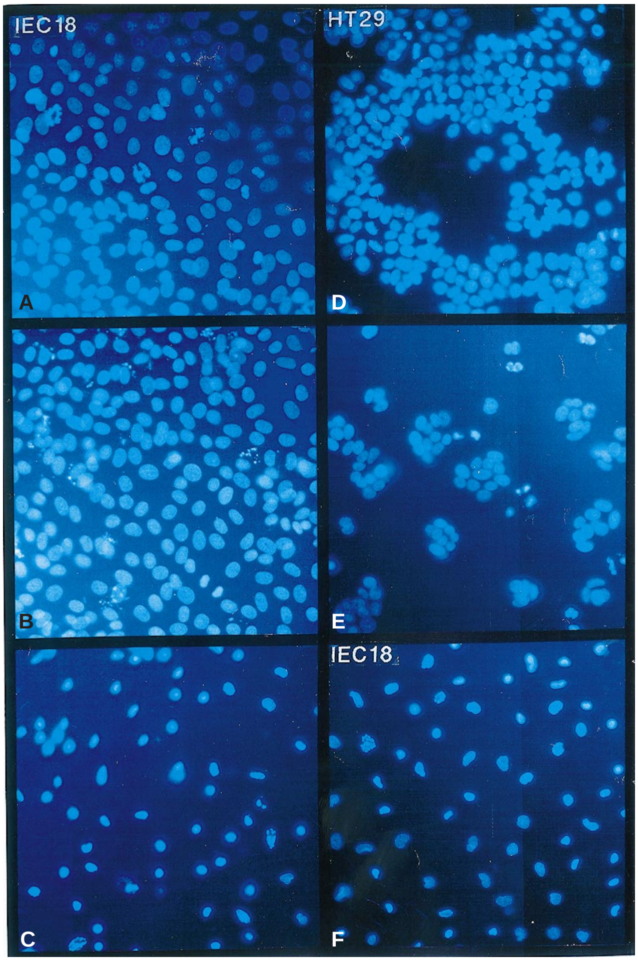

**Figure 2** (**A–C**) Hoescht labelling of IEC18 cells growing in 2% FCS and treated with 25 µg ml$^{-1}$ genistein. Control cells (**A**) growing in culture and displaying several mitotic figures gradually begin to apoptose within 24 h (**B**) until after 72 h (**C**) most cells have died and detached from the substrata. HT29 cells behave similarly: control (**D**), 72 h (**E**). IEC18 cells treated for 72 h with isoflavone 4 again appeared to die via apoptosis (**F**)

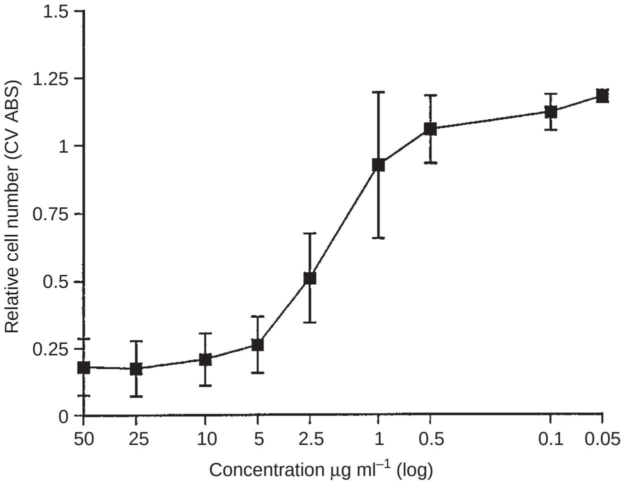

**Figure 1** Dilution curve for genistein when applied to IEC18 cells growing in 2% FCS, presented on a log scale. The cell numbers (as measured by crystal violet dye binding) after 5 days exposure, relative to the untreated control, are shown. Genistein can be seen to reduce cell number by up to 80%

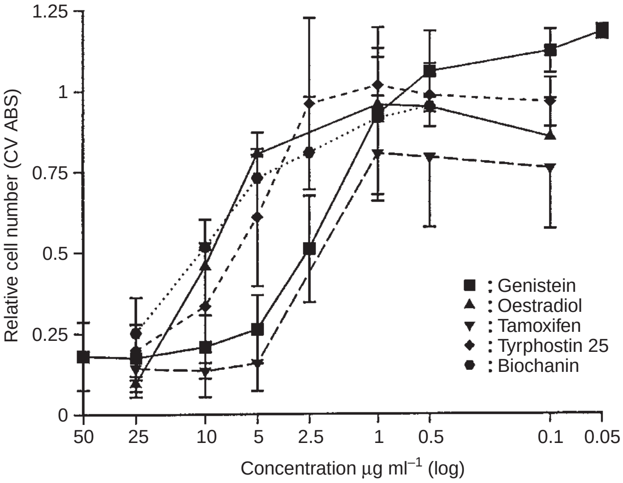

**Figure 3** Genistein and tamoxifen were potent inhibitors of IEC18 cell proliferation. Oestradiol, tyrphostin 25 and biochanin were less inhibitory, all having similar potencies

*© 1999 Cancer Research Campaign*

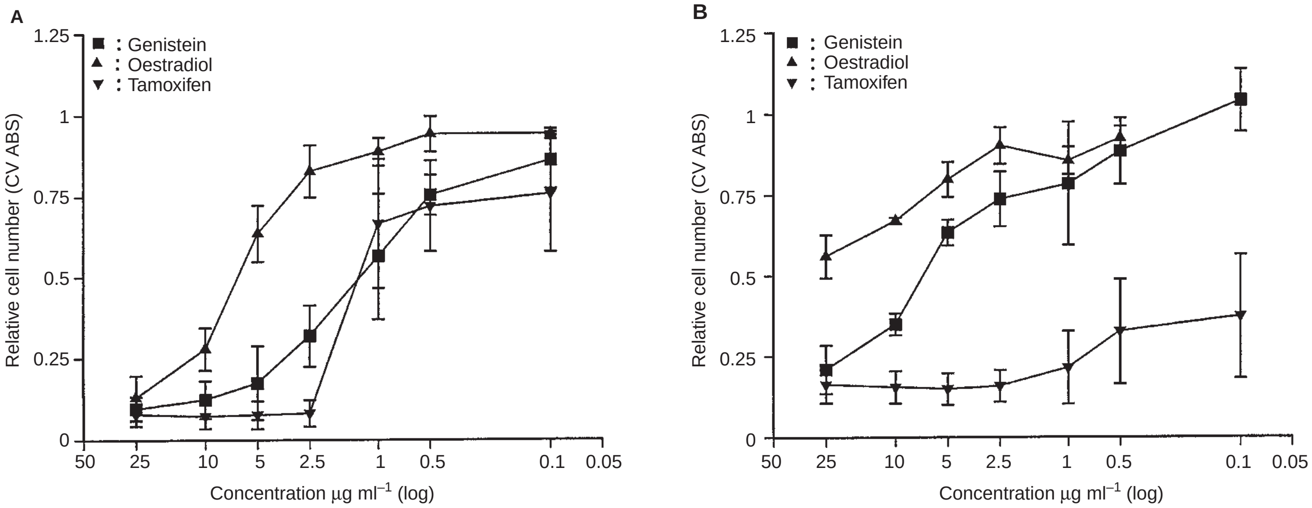

**Figure 4** Effect of genistein, oestradiol and tamoxifen on two carcinoma cell lines. Genistein exhibited similar inhibitory activity on both cell lines (SW620 cells (A) appeared slightly more sensitive). Oestradiol was less potent and but again appeared to exhibit more inhibition on the SW620 cells. Conversely, tamoxifen was much more effective on the HT29 cells, (B) with marked sensitivity (75% fewer cells) still evident in this cell line at 1 µg ml$^{-1}$, a concentration at which only 25% fewer SW620 cells were present

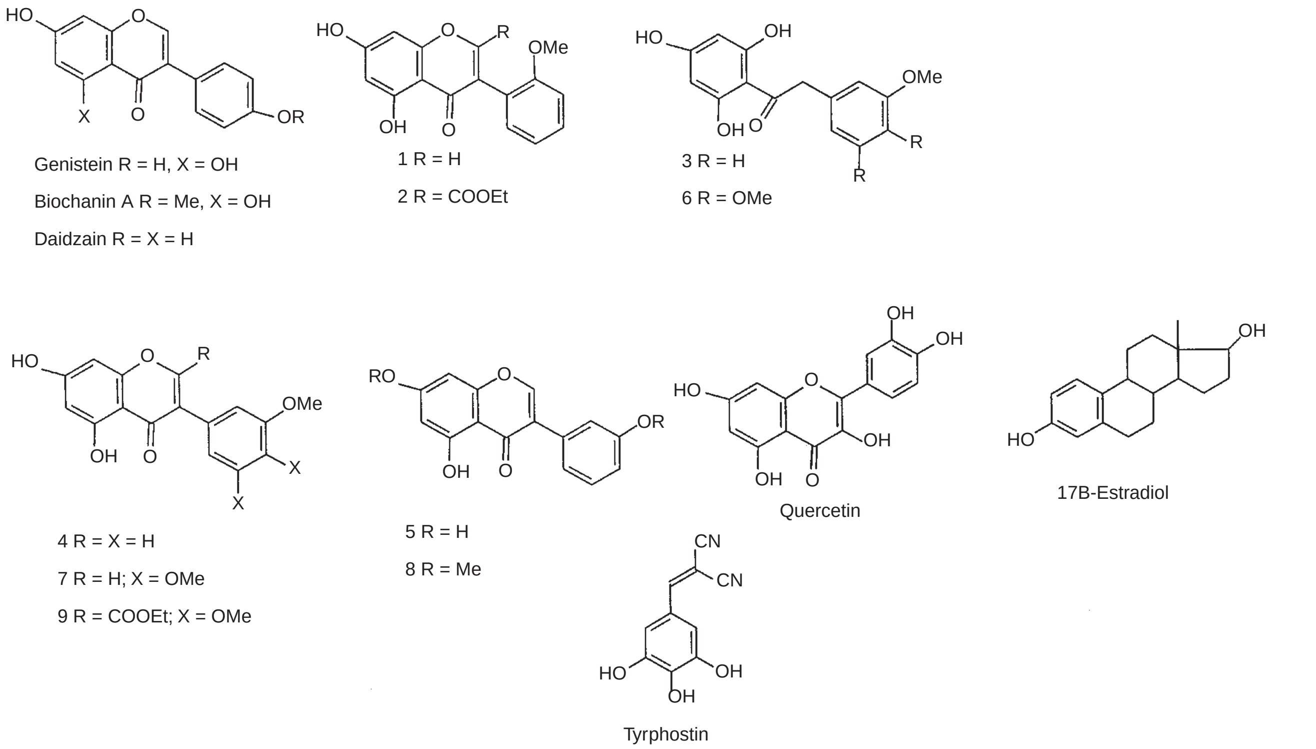

**Figure 5** Structures of the synthetic isoflavones and ketones compared to their naturally occurring analogues

### Genistein is a more potent inhibitor of intestinal cell proliferation than a range of novel isoflavones

A number of novel isoflavones were synthesized in order to try to identify potentially more potent inhibitors of cell growth (Figure 5). Their effects were assessed on both normal and transformed cell lines and compared to their natural counterparts. It can be seen from Figure 6 that all the novel isoflavones inhibited cell proliferation. The molecular weights of these compounds are all very similar, and hence results are presented as µg ml$^{-1}$ comparisons. Only isoflavone 4 approached the potency of genistein (and also killed the cells by apoptosis) (Figures 6 and 2). The relative IEC18 cellularities after exposure to 25 µg ml$^{-1}$ of isoflavones can be seen clearly in Figure 7. The same relative activities displayed in the normal intestinal epithelial cell lines (IEC6, IEC18) were also displayed in the SW620 and HT29 adenocarcinoma cell lines (Figure 8).

 *© 1999 Cancer Research Campaign*

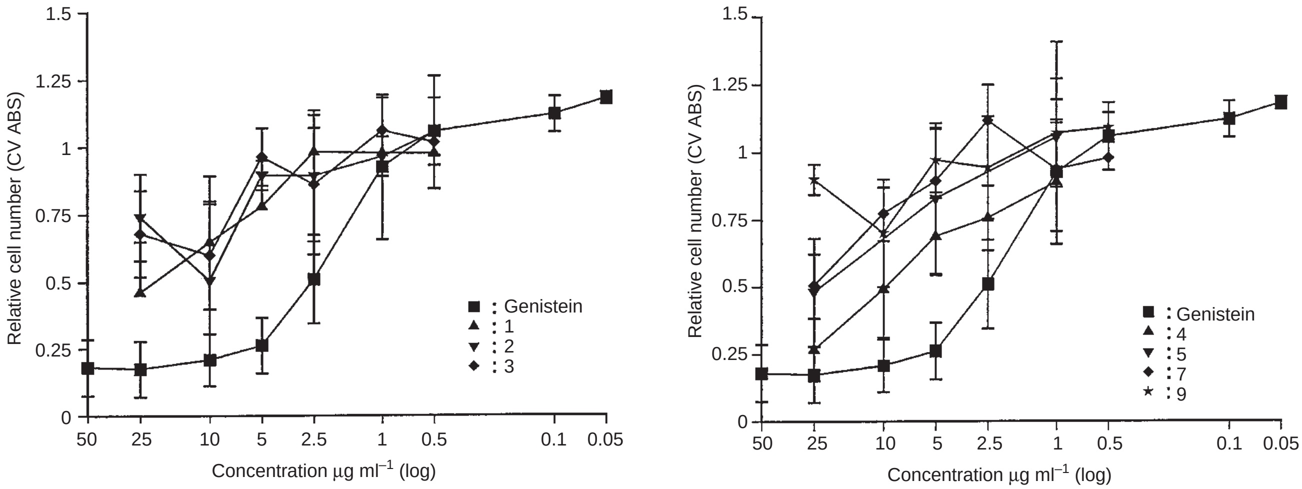

**Figure 6** Dilution curves for the synthetic isoflavones (cell numbers present after 5-day exposures). IEC18 cells were more sensitive to inhibition by genistein than any of novel isoflavones. Isoflavone 4 was the most potent synthetic analogue

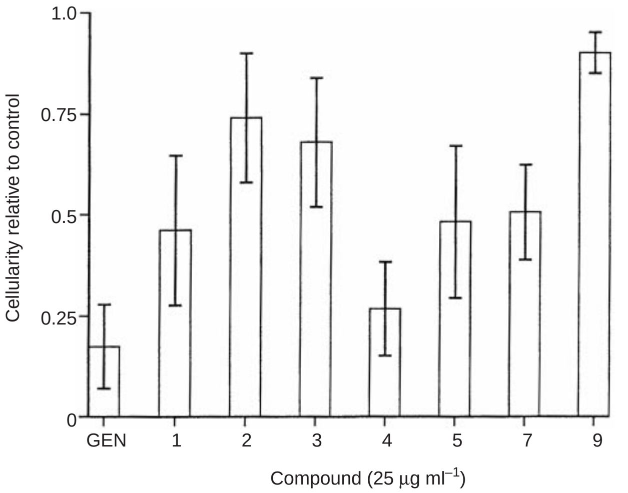

**Figure 7** After 5 days in culture at 25 µg ml$^{-1}$ genistein caused the greatest loss in cellularity (crystal violet staining), although isoflavone 4 was very similar. Isoflavones 1, 5 and 7 were equipotent causing approximately a 50% reduction in cell number. Least effective were isoflavones 2, 3 and 9

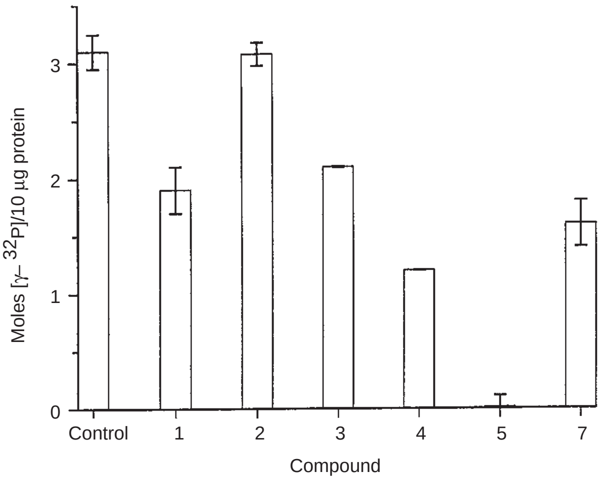

**Figure 9** Tyrosine kinase activity after exposure to synthetic isoflavones (5 mM). Activities are measured by comparing $^{32}$P radioactivity with a control sample

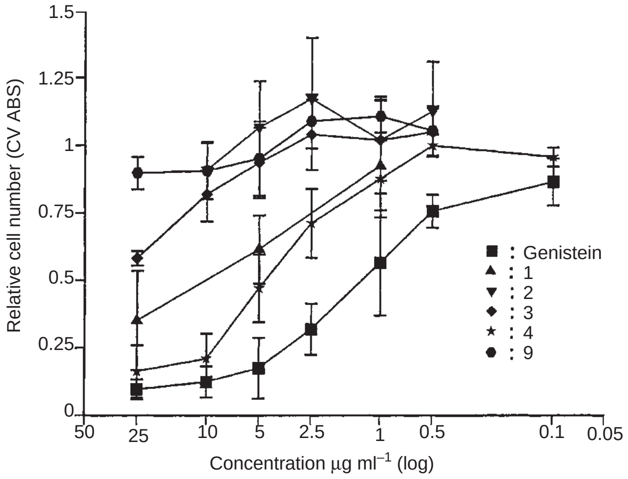

**Figure 8** SW620 cells exhibited the same sensitivities to the synthetic analogues as the IEC18 cells, i.e. genistein > 4 > 1 > 2, 3, 9

In order to test whether this result might be due to tyrosine kinase inhibition the effects of the synthetic isoflavones on the activity of this enzyme were also examined. It can be seen that a similar potency spectrum is apparent (as compared to IEC18 growth inhibition) for all the isoflavones except 5, indicating this might contribute to the mechanism of action of these isoflavones (Figure 9). A correlation of cellularity with inhibition of PTK activity showed a correlation coefficient of 0.68, although this did not attain significance ($P$ = 0.94, Spearman test). Further work on other analogues is necessary to fully evaluate the inter-relationship of these parameters.

### DISCUSSION

We have demonstrated that the isoflavones biochanin A, genistein and the synthetic analogues have an inhibitory effect on both rat intestinal (‘normal’) and human colon adenocarcinoma cell lines. Unfortunately, same species comparisons are not possible as

*© 1999 Cancer Research Campaign*

normal human or rat tumour cell lines are not available. Hence, the best available alternatives were used – cell lines that have been widely used by many groups to successfully detect normal vs tumour differences in the intestinal epithelium. We have also recently tested the effects of these compounds on a novel primary culture model of mouse colonic epithelium (Booth et al, 1995), as an additional control, with the same spectrum of activities across the isoflavones as observed in the cell lines.

The naturally occurring genistein was found to have the most potent dose-dependent effect, even compared to synthetic isoflavones synthesized particularly for their possible growth inhibitory effects. The isoflavones **1, 2, 4, 5, 7, 9** and the ketone **3** (the synthetic precursor of **4** and **5**) were synthesized as structural analogues of the naturally occurring genistein and biochanin A. Genistein is a known PTK inhibitor (Peterli et al, 1992) and biochanin A is a non-specific inhibitor of protein kinases (Wang et al, 1997). The oxygenation pattern on the phenyl ring was altered to discover whether simple isomeric alteration (**1, 2, 4, 5**) or extra methoxylation (**7, 9**) would affect the biochemistry of the isoflavones. It appears from these studies that these structural alterations do not lead to an increase in the efficacy of isoflavone-induced cell death. No structure–function relationship could be detected.

Although the exact mechanisms of action of the isoflavones have yet to be fully elucidated, induction of apoptosis may be partly responsible. Induction of apoptosis by genistein has previously been demonstrated in human cancer cells of gastrointestinal origin and it was suggested that inhibition of cell growth was via this process (Yanagihara et al, 1993). We have also shown that genistein (together with biochanin A and the other novel isoflavones) caused apoptosis in both the small intestinal and colonic cell lines, although this has not been quantitated. In addition to inducing apoptosis, genistein and the other novel isoflavones assayed exhibited tyrosine kinase inhibitory activity. Although this is a lysate rather than a cellular assay the result is consistent with other observations that have previously demonstrated genistein to be an effective epidermal growth factor receptor-associated PTK inhibitor (Akiyama et al, 1987), presumably blocking the cell proliferation signalling process. Indeed, in our study addition of the specific tyrosine kinase inhibitor tyrphostin to cells originating from both the small intestine and colon inhibited cell growth. An increase in cell proliferation is believed to be an important factor in the development of some human tumours and therefore inhibition of PTK activity has been put forward as a target for cancer therapy (Powis and Kozikowski, 1991). For example, in vivo data suggest that treatment with an epidermal growth factor-specific PTK inhibitor can inhibit mammary and oesophageal tumour growth (Toi et al, 1990). However, it is unlikely that PTK inhibition is solely responsible for the growth inhibition observed in this study, since isoflavone **5** was shown to have the highest PTK inhibitory effect, but was a less potent growth inhibitor.

The only data available regarding possibly protective levels of genistein in humans found that the peak serum concentrations in experimental subjects consuming soy milk, containing 50 mg genistein daily, reached 0.2 $\mu$g ml$^{-1}$ (Xu et al, 1994). It has also been estimated that in populations consuming soy-rich diets, serum genistein is unlikely to rise above 0.85–1.14 $\mu$g ml$^{-1}$ (Barnes et al, 1995). Our data suggest that genistein levels above 1 $\mu$g ml$^{-1}$ have an inhibitory effect upon intestinal cells, but lower levels either have no effect or stimulate cell growth. Although it is difficult to extrapolate in vitro data to the in vivo situation, the available data suggest that it may be difficult to obtain sufficient genistein to obtain beneficial biological effects from diet alone. However, the effect of long-term persistent exposure to low doses of genistein and the different sensitivities of the cells in intestinal epithelial cell hierarchy (particularly the sensitivity of the stem cells) remains unknown.

Oestradiol and tamoxifen inhibited all the cell lines. Although the intestine is a non-target organ for oestrogen, functional oestrogen receptors have been located on non-transformed intestinal cells (Thomas et al, 1993). The results of previous studies examining the effect of oestradiol upon cells of the colon have been disparate. Oestradiol has been shown to both inhibit and increase cell proliferation (Xu and Thomas, 1994; Singh et al, 1997). In humans, the use of oestrogenic hormone replacement therapy is associated with a decreased colon cancer risk (Potter et al, 1996; Kampman et al, 1997) suggesting that oestrogen may exert a protective effect. Our data support the inhibitory effect of oestradiol upon intestinal cells and suggest such protection may occur via induction of apoptosis. Phyto-oestrogens have a structural similarity to oestradiol and have been shown to have oestrogen antagonist (Pagliacci et al, 1994) and agonist (Welshons et al, 1987; McMichael-Phillips et al, 1996) activities, which is probably a dose-dependent effect (Zava et al, 1997). In breast cancer cells genistein can compete with oestradiol for oestrogen receptor binding sites (Martin et al, 1978) and the ability to process nuclear receptors and increase cell growth is analogous to the biological actions of oestradiol. It is possible that the inhibitory and stimulatory effects of genistein and the novel isoflavones assayed upon intestinal cells are due in part to these dose-dependent oestrogenic activities. Details of the effects of these compounds on oestrogen receptor binding affinity remain to be determined.

Consumption of isoflavone-rich foods, such as soya products, have been linked with a decrease in breast cancer risk (Lee et al, 1995). Furthermore, men with a low risk of prostate cancer have a higher urinary excretion of phyto-oestrogens than those populations with an increased cancer risk (Adlercreutz et al, 1993). Urinary phyto-oestrogen levels are also decreased in women with breast cancer (Ingram et al, 1997). Soyabean diets and individual isoflavones have also been shown to offer protection against mammary tumour development in animal models of cancer (Troll et al, 1980; Lamartiniere et al, 1995) and the related, naturally occurring flavone, quercetin, has been shown to decrease the growth of gastric cancer cells (Yoshida et al, 1990). It is likely that several anticancer mechanisms are involved in the protection offered by dietary isoflavones, examples that we have shown here are that apoptosis, oestrogenicity and tyrosine kinase inhibition may play a role. Genistein has also been shown to have other biological activities which may inhibit tumour formation and growth, including inhibition of topoisomerase (Okura et al, 1988) and angiogenesis (Fotsis et al, 1993) and induction of cell differentiation (Constantinou et al, 1990). However, all of these may be a direct consequence of tyrosine kinase inhibition.

Our data corroborate the previous findings that isoflavones may have protective effects and show that naturally occurring genistein is a more potent inhibitory agent than the synthetic isoflavones produced, which had been formulated specifically for their tyrosine kinase activity. Our data provide evidence that isoflavones can reduce gastrointestinal cell proliferation and induce apoptosis. An effect was observed in both non-transformed and cancer cells.

*© 1999 Cancer Research Campaign*

Therefore, isoflavones may have a role to play in both protecting normal tissues against tumour development and inhibiting established tumour growth.

### ACKNOWLEDGEMENTS

This work was carried out at the Paterson Institute for Cancer Research, Christie Hospital, Manchester. The authors would like to thank Julie A O'Shea, Kelly Middlehurst, Nita Tsui and Simon Freeman for their technical assistance. CB, JH, ATM and CSP are funded by the Cancer Research Campaign, DFH by MAFF.

### REFERENCES

Adlercreutz H (1990) Western diet and Western diseases: some hormonal and biochemical mechanisms and associations. *Scand J Clin Lab Invest* **50**: 3–23

Adlercreutz H, Markkanen H and Watanabe S (1993) Plasma concentrations of phyto-oestrogens in Japanese men. *Lancet* **342**: 1209–1210

Akiyama T, Ishida J, Nakagawa S, Ogawara H, Watanabe S, Itoh N, Masabumi S and Fukami Y (1987) Genistein, a specific inhibitor of tyrosine-specific protein kinases. *J Biol Chem* **25**: 5592–5595

Armstrong B and Doll R (1975) Environmental factors and cancer incidence and mortality in different countries, with special reference to dietary practices. *Intl J Cancer* **15**: 617–631

Barnes S, Peterson G and Coward BS (1995) Rationale for the use of genistein-containing soy matrices in chemoprevention trials for breast and prostate cancer. *J Cell Biochem* **22**: 181–187

Booth C et al (1995) The isolation and culture of adult mouse colonic epithelium. *Epithelial Cell Biol* **4**: 76–86

Boyle P, Zaridze DG and Smans M (1985) Descriptive epidemiology of colorectal cancer. *Intl J Cancer* **36**: 9–18

Casnellie J, Harrison M, Pike L, Hellstrom K and Krebs E (1982) Phosphorylation of synthetic peptides by a tyrosine protein kinase from the particulate fraction of a lymphoma cell line. *Proc Natl Acad Sci USA* **79**: 282–286

Constantinou A, Kiguchi K and Huberman E (1990) Induction of differentiation and DNA strand breakage in human HL-60 and K-562 leukemia cells by genistein. *Cancer Res* **50**: 2618–2624

Fotsis T, Pepper M, Aldercreutz H, Fleischmann G, Hase T, Montesano R and Schweigerer L (1993) Genistein, a dietary derived inhibitor of in vitro angiogenesis. *Proc Natl Acad Sci USA* **90**: 2690–2694

Hoff MB, Chang WWL and Mak KM (1981) Effect of estrogen on cell proliferation in colonic mucosa of the mouse. *Virchows Arch* **35**: 263–273

Ingram D, Sanders K, Kolybaba M and Lopez D (1997) Case-control study of phyto-oestrogens and breast cancer. *Lancet* **350**: 990–994

Kampman E, Potter JD, Slattery ML, Caan BJ and Edwards S (1997) Hormone replacement therapy, reproductive history, and colon cancer: a multicenter, case-control study in the United States. *Cancer Causes Control* **8**: 146–158

Kuo S (1996) Antiproliferative potency of structurally distinct isoflavanoids on human colon cancer cells. *Cancer Lett* **110**: 41–48

Lamartiniere CA, Moore J, Holland M and Barnes S (1995) Neonatal genistein chemoprevents mammary cancer. *Proc Soc Exp Biol Med* **208**: 120–123

Lee HP, Gourley L, Duffy SW, Esteve J, Lee J and Day NE (1995) Dietary effects on breast-cancer risk in Singapore. *Lancet* **337**: 1197–1200

Martin PM, Horwitz KB, Ryan DS and McGuire WL (1978) Phytoestrogen interaction with estrogen receptors in human breast cancer cells. *Endocrinology* **103**: 1860–1867

McMichael-Phillips DF, Harding C, Morton M, Howell A, Potten CS and Bundred NJ (1996) The effects of soy supplementation on epithelial proliferation in the normal human breast. *Breast Cancer Res Treat* **41**: 263

Messina MJ, Persky V, Setchell KDR and Barnes S (1994) Soy intake and cancer risk: a review of the in vitro and in vivo data. *Nutr Cancer* **21**: 113–131

Negi RKS, Rajagopalan TR and Batra V (1985) Flavanoidal constituents of *Lupinus hirsutus*. *Indian J Chem* **24B**: 221

Ogawara H, Akiyama T, Watanabe S, Ito N, Kobori M and Seoda Y (1989) Inhibition of tyrosine protein kinase activity by synthetic isoflavones and flavones. *J Antibiot Tokyo* **42**: 340–343

Okura A, Arakawa H, Oka H, Yoshinari T and Monden Y (1988) Effect of genistein on topoisomerase activity and on the growth of [val12] Ha-*ras*-transformed NIH 3T3 cells. *Biochem Biophys Res Comm* **157**: 183–189

Pagliacci MC, Smachia M, Migliorati F, Grignani F, Riccardi C and Nicoletti I (1994) Growth-inhibitory effects of the natural phytoestrogen genistein in MCF-7 human breast cancer cells. *Eur J Cancer* **30A**: 1675–1682

Peterli F, Stumpf R, Schweizer M, Sequin U, Mett H and Praxler P (1992) Nitrostyrene derivatives of adenosine 5′-glutarates as selective inhibitors of the epidermal growth factor receptor protein tyrosine kinase. *Helv Chim Acta* **75**: 696–706

Potten CS, Booth C, Chadwick CA and Evans GS (1993) A potent stimulator of small intestinal cell proliferation extracted by simple diffusion from intact irradiated intestine: in vitro studies. *Growth Factors* **10**: 53–61

Potter JD, Bostick RM, Grandits GA, Fosdick L, Elmer P, Wood J, Grambasch P and Louis TA (1996) Hormone replacement therapy is associated with lower risk of adenomatous polyps of the large bowel: the Minnesota Cancer Prevention Research Unit Case-Control Study. *Cancer Epidemiol Bio Prev* **5**: 779–784

Powis G and Kozikowski A (1991) Growth factor and oncogene signalling pathways as targets for rational anticancer drug development. *Clin Biochem* **24**: 385–397

Quaroni A and May RJ (1980) Establishment and characterisation of intestinal epithelial cell cultures. *Methods Cell Biol* **21B**: 403–427

Severson RK, Nomura AMY, Grove JS and Stemmermann GN (1989) A prospective study of demographics, diet and prostate cancer among men of Japanese ancestry in Hawaii. *Cancer Res* **49**: 1857–1860

Singh S, Paraskeva C, Gallimore PH, Sheppard MC and Langman MJ (1994) Differential growth response to oestrogen of premalignant and malignant colonic cell lines. *Anticancer Res* **14**: 1037–1041

Thomas MLT, Xu X, Norfleet AM and Watson CS (1993) The presence of functional estrogen receptors in intestinal epithelial cells. *Endocrinology* **132**: 426–430

Toi M, Mukaida H, Wada T, Hirabayashi N, Toge T, Hori T and Umezawa K (1990) Antineoplastic effect of erbstatin on human mammary and esophageal tumors in athymic nude mice. *Eur J Cancer* **26**: 722–724

Troll W, Wiesner R, Shellabarger CJ, Holtzman S and Stone JP (1980) Soybean diet lowers breast tumor incidence in irradiated rats. *Carcinogenesis* **1**: 469–472

Wang BH, Ternai B and Poly G (1997) Specific inhibition of cyclic AMP-dependent protein kinase by warangalone and robustic acid. *Phytochemistry* **44**: 787–796

Wang H and Murphy PA (1994) Isoflavone content in commercial soybean foods. *J Agric Food Chem* **42**: 1666–1673

Wei H, Wei L, Frenkel, Bowen R and Barnes S (1993) Inhibition of tumour promoter-induced hydrogen peroxide formation in vitro and in vivo by genistein. *Nutr Cancer* **20**: 1–12

Welshons WV, Murphy CS, Koch R, Calaf G and Jordan VC (1987) Stimulation of breast cancer cells in vitro by the environmental estrogen enterolactone and the phytoestrogen equol. *Breast Cancer Res Treat* **10**: 169–175

Willett WC (1995) Diet, nutrition and avoidable cancer. *Environmental Health Perspectives* **103**: 165–170

Xu X and Thomas ML (1994) Estrogen receptor mediated direct stimulation of colon cell growth in vitro. *Mol Cell Endocrinol* **105**: 197–201

Xu X et al. (1994) Daidzein is a more bioavailable soymilk isoflavone than is genistein in adult women. *J Nutr* **124**: 825–832

Yanagihara K, Ito A, Toge T and Numoto M (1993) Antiproliferative effects of isoflavones on human cancer cell lines established from the gastrointestinal tract. *Cancer Res* **53**: 5815–5821

Yoshida M, Sakai T, Hosokawa N, Marui N, Matsumoto K, Fujioka A, Nishino H and Aoike A (1990) The effect of quercetin on cell cycle progression and growth of human gastric cancer cells. *FEBS Lett* **260**: 10–13

Zava DT and Duwe G (1997) Estrogenic and antiproliferative properties of genistein and other isoflavonoids in human breast cancer cells in vitro. *Nutr Cancer* **27**: 31–40

© 1999 Cancer Research Campaign
